# A solitary hyperfunctioning thyroid nodule harboring thyroid carcinoma: review of the literature

**DOI:** 10.1186/1756-6614-6-7

**Published:** 2013-05-04

**Authors:** Sasan Mirfakhraee, Dana Mathews, Lan Peng, Stacey Woodruff, Jeffrey M Zigman

**Affiliations:** 1 Department of Internal Medicine, Division of Endocrinology and Metabolism, The University of Texas Southwestern Medical Center, Dallas, Texas, 75390, USA; 2 Department of Radiology, Neurology and Neurotherapeutics, The University of Texas Southwestern Medical Center, Dallas, TX, 75390, USA; 3 Department of Pathology, The University of Texas Southwestern Medical Center, Dallas, TX, 75390, USA; 4 Department of Surgery, The University of Texas Southwestern Medical Center, Dallas, TX, 75390, USA

**Keywords:** Hot nodule, Thyroid cancer, Malignancy

## Abstract

Hyperfunctioning nodules of the thyroid are thought to only rarely harbor thyroid cancer, and thus are infrequently biopsied. Here, we present the case of a patient with a hyperfunctioning thyroid nodule harboring thyroid carcinoma and, using MEDLINE literature searches, set out to determine the prevalence of and characteristics of malignant “hot” nodules as a group. Historical, biochemical and radiologic characteristics of the case subjects and their nodules were compared to those in cases of benign hyperfunctioning nodules. A literature review of surgical patients with solitary hyperfunctioning thyroid nodules managed by thyroid resection revealed an estimated 3.1% prevalence of malignancy. A separate literature search uncovered 76 cases of reported malignant hot thyroid nodules, besides the present case. Of these, 78% were female and mean age at time of diagnosis was 47 years. Mean nodule size was 4.13 ± 1.68 cm. Laboratory assessment revealed T_3_ elevation in 76.5%, T_4_ elevation in 51.9%, and subclinical hyperthyroidism in 13% of patients. Histological diagnosis was papillary thyroid carcinoma (PTC) in 57.1%, follicular thyroid carcinoma (FTC) in 36.4%, and Hurthle cell carcinoma in 7.8% of patients. Thus, hot thyroid nodules harbor a low but non-trivial rate of malignancy. Compared to individuals with benign hyperfunctioning thyroid nodules, those with malignant hyperfunctioning nodules are younger and more predominantly female. Also, FTC and Hurthle cell carcinoma are found more frequently in hot nodules than in general. We were unable to find any specific characteristics that could be used to distinguish between malignant and benign hot nodules.

## Introduction

Thyroid nodules are frequently-encountered entities in clinical practice, occurring with a prevalence of 4% by palpation [1], 33% to 68% by ultrasound examination [2,3], and 50% on autopsy series [4]. While approximately 95% of thyroid nodules are benign, certain historical, laboratory, and sonographic features raise the suspicion for malignancy [5]. As the initial step for evaluation of a thyroid nodule is measurement of serum thyroid stimulating hormone (TSH) [6,7], it is not uncommon for patients with a solitary thyroid nodule to be diagnosed with hyperthyroidism. In this setting, the thyroid nodule may represent a solitary hyperfunctioning thyroid nodule in an otherwise normal thyroid gland or it may represent a hyperfunctioning or nonfunctioning nodule occurring within a toxic multinodular goiter, within a Graves’ disease or destructive thyroiditis milieu, or in an individual with a less common cause of thyrotoxicosis [8]. Thyroid scintigraphy employs radioiodine (^123^I, ^131^I) or technetium-99m-(^99m^Tc) pertechnetate in order to differentiate these diagnostic possibilities. The distinction is important, because hyperfunctioning nodules – also referred to as “autonomous,” “autonomously-functioning,” or “hot” nodules – are thought to only rarely harbor malignancy, such that fine needle aspiration (FNA) is not traditionally indicated in this circumstance [6]. Per the 2009 revised American Thyroid Association Management Guidelines for Patients with Thyroid Nodules and Differentiated Thyroid Cancer, “Since hyperfunctioning nodules rarely harbor malignancy, if one is found that corresponds to the nodule in question, no cytologic evaluation is necessary” [6].

Here, we present the case of a woman with subclinical hyperthyroidism due to a hyperfunctioning thyroid nodule who was diagnosed with minimally-invasive follicular carcinoma after surgical resection. We also include our findings from a formal literature review on this topic, in which the historical, laboratory, and radiological features of similarly-documented cases were scrutinized to determine if there are features that differentiate hyperfunctioning thyroid carcinomas from solitary toxic adenomas. The results of a separate literature review aimed at estimating the prevalence of thyroid cancer within hot thyroid nodules are also presented. Our goal is to call attention to the fact that hyperfunctioning thyroid carcinomas are well-described in the literature (and also likely underreported), challenging the commonly-held notion that the hot thyroid nodule is very unlikely to be cancerous.

## Materials and methods

A MEDLINE literature search of English-language studies published between 1950 and January 2012 with the terms, “thyroid cancer, hyperthyroidism, surgery,” “thyroid cancer, hyperfunctioning nodule, surgery,” and “thyroid cancer, hot nodule, surgery,” was performed to determine the reported prevalence of thyroid carcinoma in patients undergoing resection of solitary hyperfunctioning thyroid nodules. Another literature search, using the terms, “hyperfunctioning thyroid carcinoma,” “toxic adenoma, thyroid carcinoma,” and “hot nodule, thyroid carcinoma” was performed using MEDLINE and by reviewing the citations of relevant articles in order to collect data on reported cases of patients with a solitary thyroid nodule found to harbor thyroid carcinoma. Case series were included provided that at least some demographic and clinical details of individual subjects were described. A third MEDLINE literature search, using the terms “hot nodule,” “hyperfunctioning thyroid nodule,” and “autonomous thyroid nodules,” was performed to establish the demographic characteristics and nodule sizes of subjects with solitary hyperfunctioning thyroid nodules. In this third group, the available studies analyzed the group of hyperfunctioning nodules en masse and did not make a distinction between hyperfunctioning nodules that were benign and those that may have been malignant. Therefore, while the aggregate data from these studies most likely involved predominantly benign cases, a small number of malignant cases also may have been included. For this reason, we will subsequently refer to this group as “predominantly-benign” hyperfunctioning (or hot) nodules.

Weighted averages of data were calculated by assigning a “weight” to each study (based on the number of subjects in the study divided by the total number of subjects for all studies), multiplying each weight by the subject mean in the corresponding study, and then taking the sum of these products.

## Results

### Patient case

A 29-year-old teacher was referred to her local endocrinologist to evaluate a palpable left thyroid nodule. While she did not report thyrotoxic symptoms at that time, she was found to have a suppressed TSH. Thyroid scintigraphy revealed a hyperfunctioning left-sided thyroid nodule, and ultrasonography revealed it to be 2.4 cm in greatest dimension, isoechoic, and in the left lower lobe. A follow-up ultrasound one year later revealed an essentially unchanged nodule measuring 2.5 cm.

The patient presented to our institution the next year, at which time she endorsed tremors, anxiety, insomnia, and oligomenorrhea. She denied local compressive symptoms, history of radiation exposure, or family history of thyroid malignancy. Her only medication was a daily multivitamin. Her weight was 106 kg with a height of 1.7 m, blood pressure of 117/73 mmHg, and heart rate of 79 beats per minute. A 1.5 cm, firm, slightly tender nodule was palpated in the left lower lobe of the thyroid; it moved with swallowing. There was no evidence of cervical lymphadenopathy or thyroid bruit. The patient had a slight, fine tremor of the hands with normal biceps deep-tendon reflexes bilaterally.

**Figure 1:**
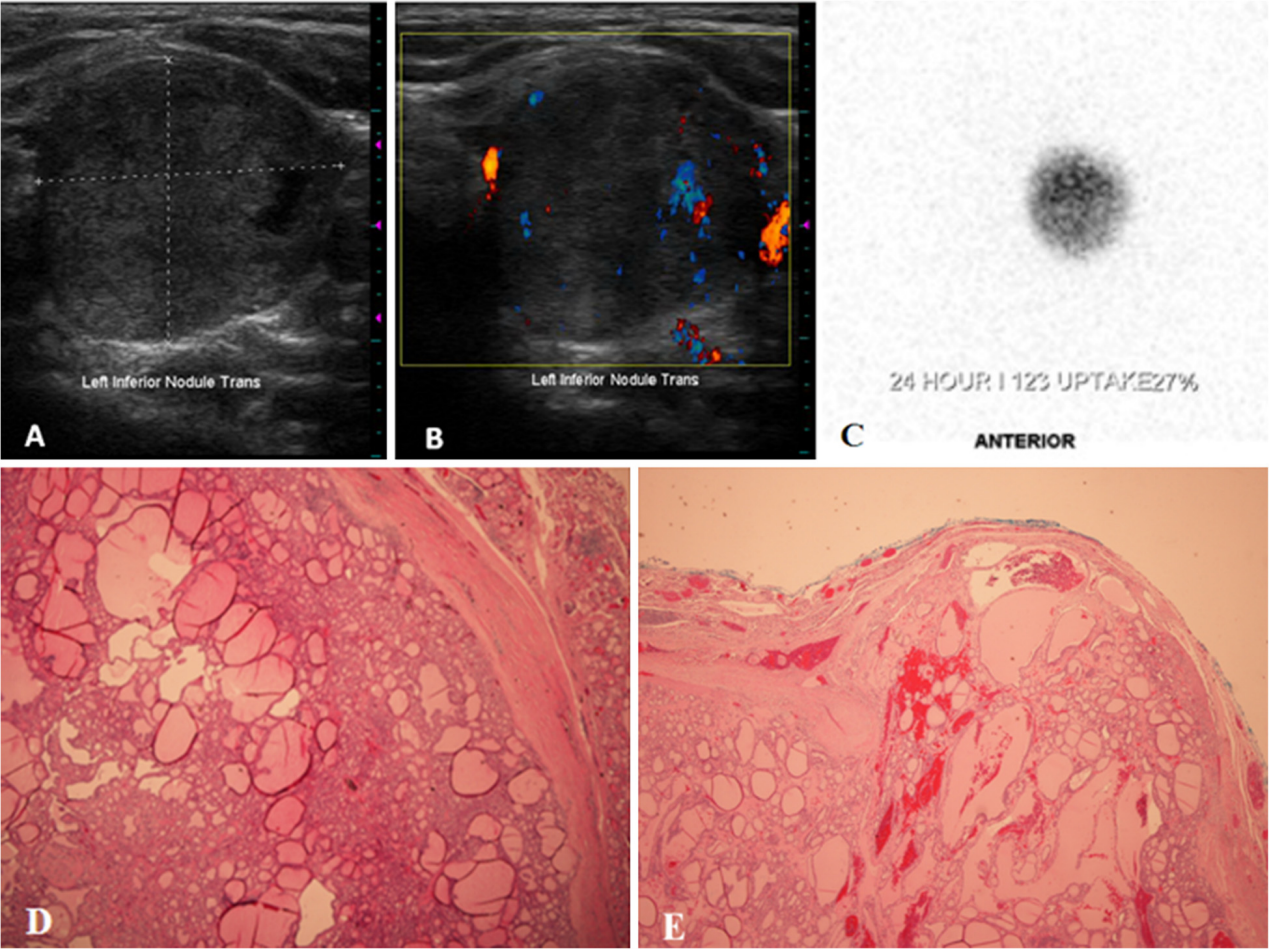
**Imaging and histologic features of the hot nodule present in the case report subject. (A)** Ultrasonography of the left thyroid lobe, demonstrating a 2.7 cm, predominantly solid, and isoechoic nodule. **(B)** Color Doppler evaluation reveals blood flow within the rim of the nodule and intraparenchymally. **(C) **^123^I thyroid scintigram depicts a round left-sided focus of iodine uptake with suppression in the remainder of the gland, consistent with an autonomously-functioning thyroid nodule. **(D)** Histological evaluation reveals that the lesion is solitary, circumscribed and encapsulated. The follicular proliferation is surrounded by a rather thick fibrous capsule. The lesion demonstrates a predominant follicular pattern of growth without papillary cytologic features (hematoxylin-eosin stain; original magnification × 4). **(E)** A focal area is identified where the tumor invades through and into the fibrous capsule (hematoxylin-eosin stain; original magnification × 2).

Thyroid function tests revealed a suppressed TSH (0.005 mcIU/mL, normal 0.4 - 4.5 mcIU/mL) but normal free T4 (1.1 ng/dL, normal 0.9 - 1.8 ng/dL) and free T3 (3.5 pg/mL, normal 2.3 - 4.2 pg/mL). Thyroid peroxidase antibody testing was negative (0.4 IU/mL, normal < 9 IU/mL), as were thyroglobulin antibody (< 20 IU/mL) and thyroid stimulating immunoglobulin (< 1.0) testing. Thyroid ultrasound revealed a 2.6 × 2.7 × 2.6 cm predominantly solid, isoechoic left lower lobe nodule with internal hypervascularity within a slightly enlarged but otherwise normal-appearing thyroid gland (Figure 1A and B). ^123^I thyroid scintigraphy revealed a left lower lobe hyperfunctioning nodule with 24-hour uptake of 27% (Figure 1C).

After discussing with the patient the benefits and risks of radioiodine ablation versus surgical resection (left hemithyroidectomy), she elected for the latter, citing the reduced risk of permanent hypothyroidism as her main deciding factor. Histologic evaluation of the surgical specimen revealed a solitary, circumscribed, and well-encapsulated tumor measuring 2.5 × 2.5 × 2.2 cm without lymphovascular invasion or extra-thyroidal extension (Figure 1D). A focus was identified where the tumor penetrated and budded through the well-defined fibrous capsule, giving the diagnosis of minimally-invasive follicular thyroid carcinoma (Figure 1E) in the setting of background lymphocytic thyroiditis. Given the pathologic diagnosis, lobectomy was felt to be sufficient, and neither completion thyroidectomy nor radioactive iodine ablation was pursued in this patient. Molecular testing was performed on the surgical specimen for BRAF and KRAS mutations, both of which were negative. At her 6-month follow up appointment, the patient was symptomatically and biochemically euthyroid on low-dose levothyroxine replacement and showed no evidence of cancer recurrence.

### Estimated prevalence of malignancy within hot thyroid nodules

In order to place the current case report in context, we have attempted to establish the prevalence of malignancy within hot thyroid nodules. This involved a literature search for surgical case series of solitary hyperfunctioning nodules managed by thyroid resection. A total of 14 relevant case series were uncovered. The earliest we found was from 1967 and included 79 hot nodules that underwent surgical resection; none of those cases were found to harbor thyroid carcinoma. Among the 14 case series, carcinoma rates of intranodular carcinoma ranging from 0 – 12.5% were noted, with a weighted total of 3.1% (Table 1). Of note, some of those case series also included cases in which thyroid carcinoma occurred outside of the hot nodule; however for the purposes of our review, such extranodular cases were not included in our estimated prevalence determination.

**Table 1:** Intranodular thyroid carcinoma prevalence in patients undergoing resection of solitary, hyperfunctioning thyroid nodules

**Author**	**Cases of hyperfunctioning nodule (n)**	**Cases of thyroid carcinoma within the hyperfunctioning nodule (n)**	**Thyroid carcinoma prevalence (%)**
**Giles **[9]	176	4	2.3
**Cakir **[10]	63	4	6.3
**Cappelli **[11]	207	3	1.4
**Foppiani **[12]	16	2	12.5
**Sahin **[13]	77	2	2.6
**Gabriele **[14]	120	2	1.7
**Harach **[15]	73	6	8.2
**Vaiana **[16]	153	3	2
**Zanella **[17]	41	3	7.3
**Terzioglu **[18]	25	2	8
**Pacini **[19]	40	1	2.5
**Smith **[20]	30	2	6.7
**Hamburger **[21]	24	1	4.2
**Horst **[22]	79	0	0
**Total**	**1124**	**35**	**3.1**

Only cases of intra-nodular malignancy are included. Studies are arranged in order of ascending date of publication.

n = number of case.

### Search for distinctive features of malignant hot thyroid nodules

We next sought to establish historical and/or clinical features that may help to differentiate malignant, hyperfunctioning thyroid nodules from benign, toxic adenomas, especially since hyperfunctioning thyroid carcinoma is generally an ex post facto diagnosis. Using the search criteria listed in the Methods Section, we discovered 76 cases (in addition to the current case) of malignant hot thyroid nodules, in which autonomy was highly suggested by both biochemical parameters of hyperthyroidism and scintigraphic evidence of increased radioiodine or labeled technetium uptake. All possibly-relevant features of the 77 cases were extracted and are displayed in Table 2, and a further analysis of these features appears in Table 3. An additional 27 cases with scintigraphic evidence suggestive of nodular autonomy but with normal thyroid function tests, absent thyroid function testing, or uninterpretable laboratory results (e.g., patients already on levothyroxine therapy at time of laboratory collection) were also found in the literature search (Table 4); these cases were excluded from subsequent analyses since autonomy seemed less certain.

**Table 2:** Reported cases of biochemically-hyperthyroid patients with a reported hyperfunctioning nodule discovered to harbor thyroid carcinoma on pathological review

**#**	**Age**	**Sex**	**Tumor growth (cm)**	**High risk history**^***a***^	**Suspicious U/S**^***b***^	**Nodule size**^***c ***^**(cm)**	**Tumor size**^***d ***^**(cm)**	**TFTs**^***e***^	**Toxic sx?**	**Compression sx?**	**Scan type**	**FNA**	**Surgical path**	**Reference (1st author)**
**1**	29	F		-	IV	2.7	2.5	SHT	+	-	^123^I		FTC	Current case
**2**	43	F		-	-	6.5	5	fT_3_,fT_4_	+	-	Tc		Hurthle	Karanchi [23]
**3**	13	F		-	IV	3.5	5	TT_3_	-	-	^123^I		Hurthle	Yalla [24]
**4**	63	M		-	-	4		fT_4_	-	-	^123^I	Suspicion of FVPTC	FVPTC	Bommireddipalli [25]
**5**	68	F		-	HE	5.3		fT_3_	+	+	Tc,^123^I		FTC	Giovanella [26]
**6**	11	F		-	-	3.5		TT_3_	-	-	^123^I	Nonspecific	PTC	Tfayli [27]
**7**	47	F		-	-	2.6	3	TT_3_,fT_4_	+	-	^131^I	PTC	FVPTC	Azevedo [28]
**8**	36	M	1.4→1.8 in 11mo	-	HE,IV	1.8	1.5	SHT	+	-	^131^I	PTC	PTC	Uludag [29]
**9**	62	F			-		2	fT_3_,fT_4_	+	-	Tc	PTC	PTC	Nishida [30]
**10**	32	M		-	-	4.3		fT_3_,fT_4_	-	-	Tc	Benign	FVPTC	Kim [31]
**11**	64	F		-		6		TT_3_	+	-	^131^I		FTC	Niepomniszcze [32]
**12**	57	F			-	6		High^*f*^	+	-	Tc	Nondiagnostic	FTC	Bitterman [33]
**13**	59	F			-	5		High	+		Tc		FTC	Bitterman [33]
**14**	59	F		-	HE,PD,Cal	1.5	1.5	fT_3_,fT_4_	+	+	Tc,^123^I	PTC	PTC	Majima [34]
**15**	NA	F				5	5	fT_3_,fT_4_	+	+	Tc		PTC	Gozu [35]
**16**	67	F				2.5	3	fT_4_	+	-	Tc	Benign	Hurthle	Wong [36]
**17**	39	F	Subjective ↑		-	2		Nl→SHT	-	- → +	^123^I		PTC	Yaturu [37]
**18**	36	M	2x size in 5yrs		-	2.8	2.3	SHT	-			Follicular neoplasm	FVPTC	Logani [38]
**19**	11	F		-	-		4	TT_3_,fT_4_	+	-	Tc,^131^I		PTC	Mircescu [39]
**20**	49	F				4	3.5	fT_4_	+	-	^123^I		FTC	Camacho [40]
**21**	47	M				3.5	3.5	fT_3_	+		^123^I	Suspicious	PTC	Bourasseau [41]
**22**	36	M				2.5	2.5	SHT			^123^I	Nondiagnostic	FTC	Bourasseau [41]
**23**	56	M				5.5	5.5	fT_4_	+		^123^I		FTC	Bourasseau [41]
**24**	39	F				1	1	fT_3_,fT_4_	+		^123^I	Suspicious	PTC	Bourasseau [41]
**25**	33	F				3	3	SHT			^123^I	Nondiagnostic	PTC	Bourasseau [41]
**26**	42	F	4.5→7.4 (no interval given)	-	-	7.4		SHT	-	-	^123^I	Benign	Hurthle	Russo [42]
**27**	17	F		-	HE	2.1	2.1	TT_3_	-	-	Tc, ^123^I		PTC	Cirillo [43]
**28**	60	F			-	5	6	TT_3_	+	-	^131^I		Insular	Russo [44]
**29**	16	F		-		2		TT_4_	+	-	^123^I	Colloid	Hurthle	Siddiqui [45]
**30**	64	F				4		High			^123^I		FTC	Mizukami [46]
**31**	25	F		-	-	4.2		TT_3_,TT_4_	+	+	^131^I		PTC/FTC	De Rosa [47]
**32**	72	M				2.8		TT_3_,TT_4_			Tc		PTC	Ikekubo [48]
**33**	52	M				5		TT_3_			Tc		PTC	Ikekubo [48]
**34**	55	F				1.6		SHT			Tc		PTC	Ikekubo [48]
**35**	67	F		-		3	2.5	TT_3_,fT_4_	+	-	^123^I	Malignant node	PTC	Sandler [49]
**36**	11	F		-	-	3.5		TT_3_	+	-	^123^I		FTC	Nagai [50]
**37**	45	F		-		3.5	3	High	+	-	^123^I		FVPTC	Nagai [50]
**38**	70	F		-		4	4	fT4	-	-	^131^I	PTC	PTC	Fukata [51]
**39**	27	F		-		3		TT_4_	+		^131^I		PTC	Sobel [52]
**40**	14	M				4		TT_3_	+	-	^131^I		PTC	Sobel [52]
**41**	32	F		-		2.5		TT_4_	+		^131^I		FVPTC	Sobel [52]
**42**	29	F		-			1	TT_4_	+	-	^131^I		PTC	Hoving [53]
**43**	44	F			irregular	2.5	0.3	TT_4_	+	-	^131^I		PTC	Khan [54]
**44**	15	F				2.5	2.5	TT_3_	+	-	^123^I		PTC	Hopwood [55]
**45**	6	F	↑ over 8mo	-		5-6x nl		High	+	-	^131^I		PTC/FTC	Sussman [56]
**46**	42	F	4→6 in 4 yrs			6	6	High		-			FTC	Dische [57]
**47**	71	F						fT_3_,fT_4_			^131^I		FTC	Als [58]
**48**	62	M				8		fT_3_			^131^I		FTC	Als [58]
**49**	62	F				7		fT_3_			^131^I		FTC	Als [58]
**50**	71	F				4		fT_3_,fT_4_			^131^I		FTC	Als [58]
**51**	69	F				6		fT_3_			^131^I		FTC	Als [58]
**52**	79	F						fT_3_,fT_4_			^131^I		FTC	Als [58]
**53**	65	M				6.5		fT_3_			^131^I		FTC	Als [58]
**54**	56	M						fT_3_			^131^I		FVPTC	Als [58]
**55**	75	M				5.5		fT_3_			^131^I		FTC	Als [58]
**56**	77	F				4		fT_3_			^131^I		PTC	Als [58]
**57**	71	F				6		fT_3_			^131^I		FVC	Als [58]
**58**	63	M				6		fT_3_			^131^I		FVPTC	Als [58]
**59**	74	F				7		fT_3_,fT_4_			^131^I		FTC	Als [58]
**60**	68	M			HE	6		SHT	-		Tc	Follicular neoplasm	FTC	Foppiani [12]
**61**	38	F			HE	2.7		SHT			Tc	Hyperplastic goiter	FTC	Foppiani [12]
**62**	35	F		-			>1cm	High	+		^131^I	PTC	PTC	Sahin [13]
**63**	65	F		-			>1cm	High	+		^131^I	PTC	PTC	Sahin [13]
**64**	19	F				5		TT_4_,TT_3_					PTC	Lin [59]
**65**	38	F		-	-		0.3	TT_3_	+		^131^I	no malignancy	PTC	Taneri [60]
**66**	44	F		-	Cal		1	TT_3_	+		^131^I	no malignancy	PTC	Taneri [60]
**67**	56	F		-			0.8	High			^131^I		FTC	Gabriele [14]
**68**	21	F		-	IV		1.6	High			^131^I		FTC	Gabriele [14]
**69**	57	F		-	-		0.7	High					PTC	Vaiana [16]
**70**	58	F		-	-		3	High					PTC	Vaiana [16]
**71**	51	F		-	-		0.6	High					PTC	Vaiana [16]
**72**	17	F		-			1	High	+				PTC	Pacini [19]
**73**	65	F	Subjective ↑			5		High	+				FTC	Terzioglu [18]
**74**	42	F						High	+				PTC	Terzioglu [18]
**75**	35	F		-		2.2	0.5	High					PTC	Zanella [17]
**76**	70	F		-		4.1	0.5	High					PTC	Zanella [17]
**77**	35	M		-		5.4	0.5	High					Hurthle	Zanella [17]

Abbreviations: + = yes; - = no; Cal = microcalcifications; FNA = fine needle aspiration; FTC = follicular thyroid carcinoma; FVPTC = follicular variant of papillary thyroid carcinoma; HE = hypoechoic; ^123^I = Iodine-123; ^131^I = Iodine-131; IV = internal vascularity; LT_4_ = levothyroxine; NA = not available; nl = normal; PD = poorly demarcated; PTC = papillary thyroid carcinoma; sx = symptoms; SHT = subclinical hyperthyroidism; fT_3_ = free triiodothyronine; fT_4_ = free thyroxine; TT_3_ = total triiodothyronine; TT_4_ = total thyroxine; ^99m^Tc = technetium-99m-pertechnetate; TFTs = thyroid function testing; U/S = ultrasound; XRT = external beam radiotherapy.

^*a*^ High-risk history: ionizing radiation exposure as child/adolescent, prior personal history of thyroid cancer, and family history of thyroid cancer in one or more 1st-degree relatives; as per Cooper et al. [6].

^*b*^ Suspicious ultrasound: hypoechoic, microcalcifications, increased nodular vascularity, poorly demarcated; as per Cooper et al. [6].

^*c*^ Nodule size: The largest diameter of the thyroid nodule measured by ultrasonography, or if ultrasound not available, then by palpation.

^*d*^ Tumor size: The largest diameter of the thyroid nodule measured grossly after surgical resection.

^*e*^ TFTs: Indicates which thyroid hormone values (total T3, total T4, free T_3_, and/or free T_4_) were elevated at time of presentation, as opposed to SHT or euthyroidism. Of note, for many of these cases, no mention of one or more of these four standard thyroid hormone values was included.

^*f*^ High: Indicates that the patient was biochemically hyperthyroid, though specific thyroid hormone levels were not given.

**Table 3:** Demographic and clinical characteristics of the reported cases of hyperthyroid patients with hyperfunctioning thyroid carcinoma from the literature and the current case (n = 77)

**Characteristic**	
Age	
Mean—yr	47.0 ± 19.8*
Distribution – no. (%)	
< 15 yr	6 (7.9%)
15-30 yr	10 (13.2%)
31-45 yr	20 (26.3%)
46-60 yr	15 (19.7%)
> 60 yr	25 (32.9%)
Sex – no. (%)	
Female	60 (77.9%)
Male	17 (22.1%)
High risk features	
Historical – no. (%)	0 (0%)
Ultrasonographical – no. (%)	11 (36.7%)
Thyroid nodule size (via ultrasound or palpation) (cm)	4.13 ± 1.68
Thyroid carcinoma size on pathological review	
Mean (cm)	2.48 ± 1.70
No. (%) with size < 1cm	8 (20.5%)
Biochemical hyperthyroidism	
T_3_ elevated – no. (%)	39 (76.5%)
T_4_ elevated – no. (%)	27 (51.9%)
Subclinical hyperthyroidism – no. (%)	10 (13.0%)
Thyrotoxic symptoms – no. (%)	37 (78.7%)
Compression symptoms – no. (%)	5 (14.7%)
Thyroid scintigraphy	
Technetium-99m-pertechnetate – no. (%)	16 (24.2%)
Iodine-123 or −131 – no. (%)	53 (80.3%)
Fine-needle aspiration	
Benign – no. (%)	7 (30.4%)
Malignant (or suspicious findings) – no. (%)	10 (43.5%)
Follicular neoplasm – no. (%)	2 (8.7%)
Nondiagnostic sample – no. (%)	4 (17.4%)
FNA accordant with final pathological diagnosis – no. (%)	12 (60%)
FNA not accordant with final pathological diagnosis – no. (%)	9 (39.1%)
Type of thyroid carcinoma on surgical pathological review	
Follicular thyroid carcinoma	28 (36.4%)
Papillary thyroid carcinoma	44 (57.1%)
Follicular variant of papillary thyroid carcinoma	8 (18.2%)
Hurthle cell carcinoma	6 (7.8%)
Insular cell carcinoma	1 (1.3%)

* Mean ± standard deviation.

no. = Number of subjects with this characteristic, % = percentage of subjects with this characteristic.

**Table 4:** Additional cases with scintigraphic evidence suggestive of an autonomous thyroid nodule without documented hyperthyroidism (or already on levothyroxine replacement therapy) discovered to harbor thyroid carcinoma on pathologic review

**#**	**Age**	**Sex**	**Tumor growth (cm)**	**High risk history**^***a***^	**Suspicious U/S**^***b***^	**Nodule size**^***c ***^**(cm)**	**Tumor size**^***d ***^**(cm)**	**TFTs**^***e***^	**Toxic sx?**	**Compression sx?**	**Scan type**	**FNA**	**Surgical path**	**Reference (1st author)**
**1**	51	F	2.7→5.3 in 2yrs	-	-	5.3	5	on LT_4_	-	+	Tc,^131^I	Follicular neoplasm	Poor diff cancer	Low [61]
**2**	44	F		-		3.5	3.7	nl	-	- →+	Tc	Benign	FTC	Schneider [62]
**3**	47	M				1.4	1	nl	-		^123^I		PTC	Bourasseau [41]
**4**	34	F				1	1	nl	-		^123^I	“Cancer”	PTC	Bourasseau [41]
**5**	37	F				1.5	1.5	nl	-		^123^I	Nondiagnostic	FTC	Bourasseau [41]
**6**	39	M				3		nl			^123^I		FVPTC	Mizukami [46]
**7**	69	F		Prior PTC		4	3.3	on LT_4_	+	-	^131^I		Hurthle	Caplan [63]
**8**	39	M			HE,PD,Cal		1.5	nl	-	-	^123^I		PTC	Michigishi [64]
**9**	65	F				4.3		nl			Tc		PTC	Ikekubo [48]
**10**	37	F				2.5		nl			Tc		PTC	Ikekubo [48]
**11**	39	F				3.5		nl			Tc		PTC	Ikekubo [48]
**12**	38	F				4.5		nl			Tc		PTC	Ikekubo [48]
**13**	35	F		-		1	0.4	nl	-	-	^123^I		PTC	Rubenfeld [65]
**14**	51	M		XRT		“large”		on LT_4_	-		^123^I		FTC	Nagai [50]
**15**	19	F	4x2→4x3 in 1 yr			4	4	nl	-	-	^131^I		PTC/FTC	Abdel-Razzak [66]
**16**	15	F		-				nl	-	-	Tc		PTC	Scott [67]
**17**	27	F			-	4	2.3	nl	+	-	^131^I		PTC	Fujimoto [68]
**18**	21	F				3	1	NA	+	+	^131^I		PTC	Becker [69]
**19**	23	F				1.5	1	NA	-	-	^131^I		PTC	Becker [69]
**20**	28	M				4.5	0.5	NA	+	-	^131^I		PTC	Molnar [70]
**21**	54	M				8.5		NA			^131^I		FTC	Als [58]
**22**	62	F						NA			^131^I		PTC	Als [58]
**23**	61	M						NA			^131^I		FTC	Als [58]
**24**	50	M				10		NA			^131^I		FTC	Als [58]
**25**	65	F				5		NA			^131^I		FTC	Als [58]
**26**	55	F				5.5		NA			^131^I		FTC	Als [58]
**27**	66	F			Cal			nl	-	+	Tc,^131^I	Colloid goiter	PTC	Bitterman [33]
**77**	35	M		-		5.4	0.5	High^*f*^					Hurthle	Zanella [17]

Abbreviations: + = yes; - = no; Cal = microcalcifications; FNA = fine needle aspiration; FTC = follicular thyroid carcinoma; FVPTC = follicular variant of papillary thyroid carcinoma; HE = hypoechoic; ^123^I = Iodine-123; ^131^I = Iodine-131; IV = internal vascularity; LT_4_ = levothyroxine; NA = not available; nl = normal; PD = poorly demarcated; PTC = papillary thyroid carcinoma; sx = symptoms; SHT = subclinical hyperthyroidism; fT_3_ = free triiodothyronine; fT_4_ = free thyroxine; TT_3_ = total triiodothyronine; TT_4_ = total thyroxine; ^99m^Tc = technetium-99m-pertechnetate; TFTs = thyroid function testing; U/S = ultrasound; XRT = external beam radiotherapy.

^*a*^ High-risk history: ionizing radiation exposure as child/adolescent, prior personal history of thyroid cancer, and family history of thyroid cancer in one or more 1st-degree relatives; as per Cooper et al. [6].

^*b*^ Suspicious ultrasound: hypoechoic, microcalcifications, increased nodular vascularity, poorly demarcated; as per Cooper et al. [6].

^*c*^ Nodule size: The largest diameter of the thyroid nodule measured by ultrasonography, or if ultrasound not available, then by palpation.

^*d*^ Tumor size: The largest diameter of the thyroid nodule measured grossly after surgical resection.

^*e*^ TFTs: Indicates which thyroid hormone values (total T3, total T4, free T_3_, and/or free T_4_) were elevated at time of presentation, as opposed to SHT or euthyroidism. Of note, for many of these cases, no mention of one or more of these four standard thyroid hormone values was included.

^*f*^ High: Indicates that the patient was biochemically hyperthyroid, though specific thyroid hormone levels were not given.

#### Demographic characteristics

We compared age at diagnosis and female: male ratio of individuals with malignant, hyperfunctioning thyroid nodules to those with benign, toxic adenomas. Data on the latter group was compiled from a separate literature search which identified several surgical case series of predominantly-benign hot nodules, as described in the Methods section (Table 5). Subjects with malignant hot nodules were younger at the time of diagnosis than those listed in multiple case series of predominantly-benign hot nodules [47.0 vs. 57.6 years, respectively (Tables 3 and 5)]. Additionally, a greater percentage of subjects with malignant hot nodule were female as compared to those with predominantly-benign hot nodules [3.53:1 vs. 1.65:1 female to male ratio, respectively (Tables 3 and 5)].

**Table 5:** Demographic characteristics of patients with solitary hyperfunctioning thyroid nodules

**Author**	**Mean age (years)**	**Female: male (n)**
**Bransom **[71]	49.7±13.4	33:35
**Blum **[72]	50.9±17.5	31:4
**Burch **[73]	45.8±16.8	42:13
**Cappelli **[11]	61.8	445:381
**Giles **[9]	48.5±11.4	147:29
**Hamburger **[21]	60±15	19:5
**Iwata [**[74]	48.8±15.4	44:0
**Landgarten **[75]	49.3	106:11
**Linos **[76]	40	53:9
**Sahin **[13]	58.3±13.7	50:27
**Smith **[20]	34.8	24:6
**Vaiana **[16]	61.8	340:290
**Weighted average**	**57.6**	**1.65:1**

#### Size

**Figure 2:**
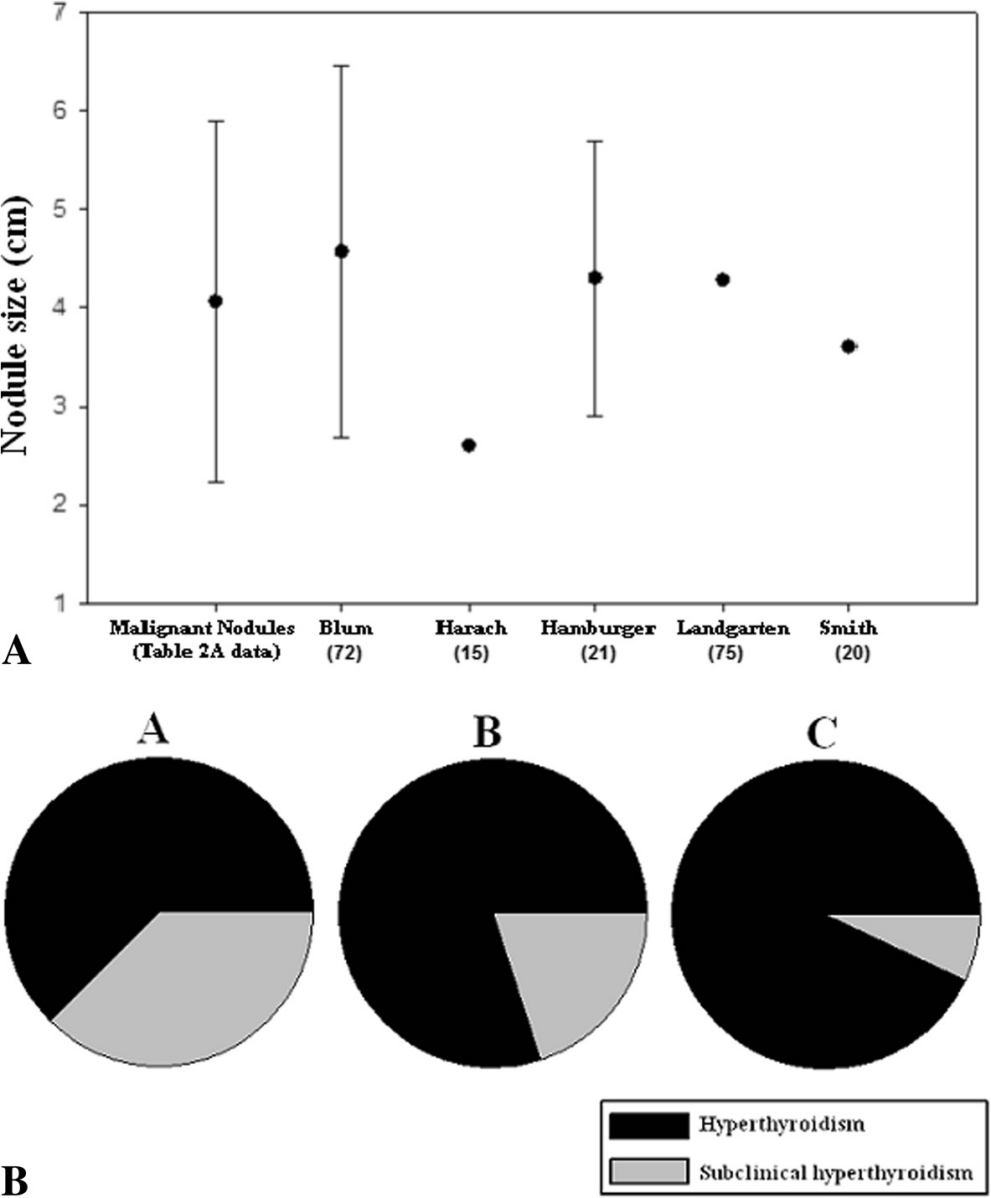
**Size and biochemical assessment of hyperfunctioning thyroid nodules.** (**A**) The mean greatest dimension of the malignant hot thyroid nodules from our case series is compared with that from five published surgical cases series of solitary, hyperfunctioning thyroid nodules. (**B)** The proportion of subjects with scintigraphically-determined hyperfunctioning thyroid carcinoma (Tables 2) who have frank biochemical hyperthyroidism vs. subclinical hyperthyroidism, based on varying nodule size. Subjects are characterized as having nodules < 2.5 cm (**A**), 2.5 – 4.5 cm (**B**), and > 4.5 cm (**C**) in diameter.

The sizes of the malignant hot nodules (Table 2) and predominantly-benign hot thyroid nodules are compared in Figure 2A. Of note, the clinical use of ultrasonography began in the late 1960s, so that the sizes listed in the early case reports of malignant hot nodule were estimated by palpation. We include tumors that were noted to encompass the entirety of the hot nodule as well as microcarcinomas embedded within a larger hot nodule. The actual size of the tumor within the thyroid nodule determined by pathological review was occasionally provided in the literature and is listed in Tables 2 and 4. The mean nodule size among the subjects with thyroid carcinoma was 4.13 ± 1.68 cm, which closely approximates the mean size of the predominantly-benign hyperfunctioning nodules.

#### Biochemical profile

The majority of subjects with a malignant hot nodule demonstrated elevation of triiodothyronine (76.5%), whereas closer to half of the subjects had elevation of thyroxine (51.9%) (Table 3). Note that these percentages represent the number of subjects with an elevated thyroid hormone level (total and/or free value, depending on the particular study) divided by the total number of subjects with a hyperfunctioning thyroid nodule. Far fewer subjects had subclinical hyperthyroidism (13%) (Table 3). In comparison, with the exception of 3 studies, similar data on the biochemical profiles of subjects with benign, toxic adenoma in the collected case series were only sparsely included. In a study of 35 subjects with toxic adenoma, Hamburger found 16 with elevations in both T_3_ and T_4_ levels, 16 with elevations of only T_3_, 3 with isolated T_4_ excess, and 7 with subclinical hyperthyroidism [77]. In a study of 63 patients with solitary toxic adenoma, Langer found that mean free T_3_ level was significantly higher (8.8 ± 3.5 pg/mL, normal range 4 – 6.8 pg/mL) than mean free T_4_ level (16.9 ± 6.6 pg/mL, normal range 7 – 17.5 pg/mL) [78]. Blum evaluated 35 patients with solitary autonomous thyroid nodule and found that 65% had elevated T_3_ levels, 54 % had elevated T_4_ levels, and 31% were biochemically euthyroid [72]. Thus, hypersecretion of T_3_ appears to be a common factor among both benign and malignant hyperfunctioning thyroid nodules. Of interest, a greater prevalence of frank biochemical hyperthyroidism can be seen in patients with malignant hyperfunctioning thyroid nodules who have larger nodules (Figure 2B).

#### Historical and sonographic features

We next turned our attention to high-risk historical features and suspicious sonographic features to determine if these were present in cases of malignant hot nodule (Table 3). These features are described in Cooper et al. [6] and are listed in the Tables 2 and 3 legend. None of the 77 subjects with malignant hot nodules was noted to have high-risk historical features. Eleven subjects (36.7%) had suspicious features on ultrasonography, which is likely an underestimate, as some of the more newly-recognized high-risk sonographic features (e.g., taller than wide on transverse view) were not utilized in the earlier published reports. It was difficult to assess cases for nodule growth as a risk factor for malignancy, since the vast majority of the subjects were referred to surgery for immediate resection. However in seven cases, nodules were noted to grow over time. While an increase in nodule size is an indication for biopsy, the specificity of this finding for malignancy is limited, as 9-89% of benign nodules have been shown to grow over time depending on which definition for significant growth is used [21,73,77,79].

#### Histologic subtype

The majority of malignant hot nodules were proven to be PTC (57.1%), and of these, 18.2% were the follicular variant of PTC (FVPTC). Follicular thyroid carcinoma (as seen in our patient) comprised 36.4% of cases, while Hurthle cell carcinoma was found in 7.8% of samples. By comparison, in the U.S. National Cancer Data Base (1985–1995), which includes histologic information on all thyroid nodules as a group, the prevalence of PTC was approximately 85%, FTC was 10%, and Hurthle cell carcinoma was nearly 3% [80]. Thus, there does seem to be a higher prevalence of both FTC and Hurthle cell carcinoma in the hyperfunctioning thyroid carcinoma cases.

Of note, within the 77 identified cases of malignant hot nodule, only 23 subjects received FNA of their thyroid nodule prior to resection. FNA enabled a pre-operative diagnosis of thyroid carcinoma in 43.5% of cases. However, 30.4% of subjects undergoing FNA were erroneously characterized as having benign lesions, and 17.4% of samples were nondiagnostic. Of the subjects with false negative biopsies, surgical pathology eventually revealed PTC in 3 cases (one of these was FVPTC), FTC in 1 case, and Hurthle cell carcinoma in 3 cases. Tumor size was given in only 3 instances of false negative biopsies. Two of these were of subcentimetric PTC, and it is presumed that small size was likely a chief factor leading to misdiagnosis. However, the other was a 3 cm nodule comprised wholly of Hurthle cell carcinoma.

## Discussion

To our knowledge, the current data set is the largest and most detailed to date of patients with malignant hot thyroid nodule. In that regard, this study complements the informative and excellent 2012 review by Pazaitou-Panayiotou and colleagues examining the association of thyroid carcinoma with a broader spectrum of hyperthyroid states, including Graves’ disease and toxic multinodular goiter in addition to hyperfunctioning thyroid nodule [81]. Of note, although it was not the focus of the Pazaitou-Panayiotou et al. review to perform a detailed analysis of the historical and clinical features of malignant hot nodule cases, as we did here, Pazaitou-Panayiotou and colleagues did include an evaluation of thyroid carcinoma prevalence. The reported percentages of thyroid carcinoma in their collected case series of patients with hot nodules, which overlapped but did not mirror exactly those case series evaluated here and which also included some cases of thyroid carcinoma occurring in extranodular thyroid tissue, ranged between 2.5 – 12.0%. This corresponds to a weighted average of 6.9% and thus is similar to the 3.1% prevalence estimated here. In its discussion of thyroid carcinoma in patients with hyperfunctioning nodules, the Pazaitou-Panayiotou et al. review also included several other important commentaries. For instance, they draw attention to two studies suggestive of a higher prevalence of thyroid carcinoma in hot nodules occurring in children [82,83], though the latter study included children from an iodine-deficient region. In addition, they discuss several reports in which activating mutations of the TSH receptor gene were identified within malignant hot nodules [81].

There are several noteworthy discussion points and implications to the findings presented here. The first relates to the prevalence of malignancy within solitary hot nodules. As mentioned, in the available surgical case series that addressed this topic, a varied prevalence was noted, ranging from 0 – 12.5%, with a weighted average of 3.1%. Only a minority of the patients who underwent surgery in those series did so because of concerning findings from FNA, and as such, we do not believe that the data set is biased towards cases in which a post-operative diagnosis of malignancy was expected. We realize that this collection of case series includes only a small fraction of the total number of solitary hot nodules that have occurred, and also that the vast majority of malignant hot nodules likely have gone unreported. Furthermore, it is likely that malignancy goes undiagnosed in many cases of hot nodules treated with radioiodine, especially those harboring microcarcinomas. Despite these limitations, we do believe there are sufficient numbers of cases included among the collected 14 surgical case series of subjects to cite the 3.1% figure as being a fair and representative estimate of malignancy prevalence amongst solitary hyperfunctioning nodules. While this 3.1% prevalence figure is low – and in fact is lower than the estimated 5 - 15% prevalence of malignancy among all thyroid nodules [6] – it is not trivial. Thus, the possibility of malignancy within a hot nodule must not be overlooked by a managing clinician, particularly if management other than surgical resection is chosen.

Another important discussion point regards the limitations resulting from the retrospective nature of this analysis, including incomplete data and differing methodology used in many of the collected case reports and case series of malignant hot nodule. While some of the primary sources were meticulous in their case descriptions, others included less-thorough descriptions. For example, tumor size was frequently not reported, and thus it was unclear if a PTC tumor was simply an incidental microcarcinoma embedded within a larger hot nodule or a large, follicular variant of PTC comprising the entirety of the nodule. While we have included some cases in which the malignancy was a microcarcinoma and thus of uncertain clinical significance [84], these cases are clearly in the minority (only 8 of the 77 reported tumors were less than one centimeter in size). Other factors affecting the data set are the evolution of technology and our understanding of risk factors for thyroid carcinoma. For instance, thyroid ultrasound began clinical use in the late 1960s, and prior to this, nodule size was estimated by palpation. Earlier reports would not have commented on some of the suspicious sonographic features recognized today. Additionally, the radioimmunoassays used to measure TSH have undergone many generations of refinement over the past several decades, and thus the presence and degree of hyperthyroidism may have been underestimated in earlier studies.

Also worth discussing are the differential prevalences of thyroid carcinoma histologic subtype found in hot nodules as compared to nodules as a group. As mentioned, there was a much higher prevalence of both FTC and Hurthle cell carcinoma in hot nodules (36.4% and 7.8%, respectively) as compared to in all nodules (10% and nearly 3%, respectively). Additionally, a substantial percentage of the PTC cases found in hot nodules were the follicular variant. This may have bearing on the current algorithm for evaluation of patients with thyroid nodules as recommended in the 2009 thyroid nodule and thyroid carcinoma management guidelines [6]. In particular, for those nodules found by biopsy to have follicular neoplasm by histology, the guidelines recommend consideration be made for performing an ^123^I thyroid scan, if not already done, especially if the serum TSH is in the low-normal range; if the nodule is found to be hyperfunctioning, it can then be followed [6]. However, the high prevalence of both FTC and FVPTC reported for malignant hot nodules suggests that a biopsy diagnosis of follicular neoplasm within a hot nodule may not be as reassuring as previously thought. Also of interest, in a 2009 study, Sundaraiya and colleagues reported a case of metastatic FTC occurring in the setting of thyrotoxicosis, in which high-grade extrathyroidal uptake of technetium-99m-pertechnetate was observed; in a literature search, they found 74 other cases of thyrotoxicosis resulting from well-differentiated thyroid cancer metastatic lesions, most of which demonstrated histologic evidence of FTC [85].

As a final point of discussion, since none of the historical, biochemical or radiologic characteristics that were assessed seems to predict malignancy in the collected cases of hot nodules, one might ask if there is any utility in biopsying hot nodules. Such would be a shift from the current thyroid nodule management guidelines which views an increased nodular radiotracer uptake pattern as a reassuring characteristic from a cancer perspective [6]. Given the estimated 3.1% prevalence of malignancy within hot nodules, and also taking into account the difficulty in predicting whether a particular hot nodule is malignant, we recommend that hot nodules that are not treated surgically (as is an option to manage the hyperthyroidism) be considered for biopsy if high-risk historical and/or suspicious sonographic features are present or if these nodules grow over time, just as is currently recommended for nodules that are not hyperfunctioning [6]. This should also include previously hyperfunctioning nodules treated with radioiodine, although the known occurrence of dystrophic calcification and cystic degeneration as sequelae of radioiodine ablation should be taken into account when assessing their sonographic features [86]. Future prospective studies could help determine if biopsying at the time of diagnosis all hot nodules with suspicious sonographic characteristics and/or associated high-risk historical features versus biopsying hot nodules only if the initial sonographic characteristics worsen over time would result in better outcomes.

## Competing interests

The authors declare that they have no competing interests.

## Authors’ contributions

SM performed the literature review and drafted the manuscript. DM provided the radiology images and participated in drafting/revising the manuscript. LP provided the pathology images and participated in drafting/revising the manuscript. SW helped to provide the surgery perspective and edited the manuscript. JZ assisted with the literature review and drafting of the manuscript. All authors read and approved the final manuscript.
